# Variable Scheduling to Mitigate Channel Losses in Energy-Efficient Body Area Networks

**DOI:** 10.3390/s121114692

**Published:** 2012-11-02

**Authors:** Yuriy Tselishchev, Athanassios Boulis, Lavy Libman

**Affiliations:** 1 Networks Research Group, School of IT, The University of Sydney, Eveleigh, NSW 2006, Australia; 2 National ICT Australia, 13 Garden Street, Eveleigh, NSW 2015, Australia; 3 School of Computer Science and Engineering, The University of New South Wales, Sydney, NSW 2052, Australia; E-Mail: lavy.libman@unsw.edu.au

**Keywords:** body area networks, TDMA, energy efficiency, reliability, variable scheduling

## Abstract

We consider a typical body area network (BAN) setting in which sensor nodes send data to a common hub regularly on a TDMA basis, as defined by the emerging IEEE 802.15.6 BAN standard. To reduce transmission losses caused by the highly dynamic nature of the wireless channel around the human body, we explore *variable* TDMA scheduling techniques that allow the order of transmissions within each TDMA round to be decided on the fly, rather than being fixed in advance. Using a simple Markov model of the wireless links, we devise a number of scheduling algorithms that can be performed by the hub, which aim to maximize the expected number of successful transmissions in a TDMA round, and thereby significantly reduce transmission losses as compared with a static TDMA schedule. Importantly, these algorithms do not require *a priori* knowledge of the statistical properties of the wireless channels, and the reliability improvement is achieved entirely via shuffling the order of transmissions among devices, and does not involve any additional energy consumption (e.g., retransmissions). We evaluate these algorithms directly on an experimental set of traces obtained from devices strapped to human subjects performing regular daily activities, and confirm that the benefits of the proposed variable scheduling algorithms extend to this practical setup as well.

## 1. Introduction

Body Area Networks (BAN) are an emerging technology that has attracted attention from researchers in academia and industry due to its significant potential in many different areas including health care, sports, military, and entertainment. A typical BAN application involves a number of low-power sensing devices, operating on or around the human body, that collect sensor data, possibly processing it locally, and transmit the information to a central device, known as a *coordinator*, *sink*, or *hub*. BANs exhibit some similarities with other wireless networks, such as the master-slave structure of cellular networks and the low-power requirements of wireless sensor networks. Despite the similarities, BANs have several characteristics that require a unique approach to the design of physical layer as well as network protocols. One of these is the extreme limitations on power usage (having to last months and even years using minuscule, unobtrusive batteries), which, in combination with the short range and existence of a central hub, lead to novel power-saving techniques [1–6]. More importantly, the wireless propagation properties in BAN are quite different from many other contexts, due to the low transmission power that the nodes must use and due to the prevalent absorption effects of the human body. BANs must cope with deep fading effects that can last much longer (10–300 ms) than similar effects in cellular networks [7,8] and possible severe shadowing effects that can cause loss of connectivity up to several minutes [9]. The uniqueness and importance of these networks is reflected in the creation of the IEEE 802.15 Task Group 6 [10] that is defining the physical and MAC layer communication standards specifically for BANs.

The current draft proposal of the IEEE BAN Task Group puts forth a TDMA-based approach as the most appropriate MAC solution to achieve the desired energy efficiency [11]. Indeed, a TDMA mechanism avoids many common causes of energy waste, e.g., collisions, overhearing and idle listening, while at the same time allowing nodes to turn their radios off outside of their allocated TDMA slots, thus achieving significant energy savings. On the other hand, a simple static TDMA allocation may lead to significant waste due to the unreliable nature of wireless links around the body, namely, when a node's allocated time slot comes when its channel is in a bad state (while preventing a transmission by any other node that may have a good link). Ideally, transmission slots should be allocated to sensor nodes only when their link state to the hub allows a successful data transfer, which implies that the scheduling should not be fixed but rather vary according to the real-time link conditions of the nodes.

A TDMA mechanism typically involves splitting time into super-frames, or rounds. At the start of each round, all the nodes turn their radios on to listen for the beacon packet, transmitted by the hub to convey important network management information and assist with time synchronization. This fact can be used in implementing a variable TDMA schedule, namely, by informing all the nodes (either within the beacon packet itself or in a separate packet transmitted immediately after the beacon) about the slot allocations for the upcoming round. Thus, any beacon-based TDMA MAC protocol (including the upcoming 802.15.6 standard [11]) can support a variable slot allocation scheme.

Our contribution in this work is as follows. As a first step, we formulate the optimal variable scheduling problem based on a simple, two-state (on/off) Gilbert model of wireless links, and propose a number of scheduling strategies for the hub node based only on its observation of nodes' transmission outcomes (success or failure). Through simulation, based on Gilbert parameter ranges extracted from a set of experimental Received Signal Strength Indicator RSSI traces [12], we evaluate these strategies numerically in terms of the reduction of loss rate as compared with a static TDMA allocation, and uncover a number of important insights. Subsequently, we consider the use of the additional information that may be available from actual RSSI readings of successful transmissions (rather than simply the binary success/failure outcome), and we extend the above strategies with further heuristics that aim to capture the dynamics of RSSI fluctuations, which are shown to reduce the loss rate even further. In particular, we make the encouraging finding that near-optimal performance can be consistently attained with a simple (so-called “*Flipping*”) strategy that orders the transmissions in each round according to the order of the previous round and observed RSSI values, does not require any *a priori* knowledge of statistical parameters of each link, and is trivial to implement and compute in real time.

The rest of the paper is structured as follows. Section 2 discusses the related work, followed by Section 3 that presents our system and wireless channel model and formulates the slot scheduling problem. Sections 4 and 5 present our proposed scheduling solutions with and without the observation of actual RSSI values, respectively. Finally, Section 6 concludes the paper.

## 2. Related Work

The idea of dynamic assignment of transmission slots has been previously studied in the context of the 802.15.4 communication standard, where the network coordinator is able to vary the allocation of Guaranteed Transmission Slots (GTS) on a per-round basis and convey it to the sensor nodes in a periodic beacon packet. Initially it was noted that simple GTS allocation mechanism proposed in the standard is ineffective and leads to low bandwidth utilization due to the fact that large parts of the GTS remain unused [13,14]. It was shown that dynamic allocation of GTS on a per-round basis, where each slot is shared among several nodes, not only improves bandwidth utilization but also leads to better quality of service (QoS) and reduces the number of packets dropped due to overflow. A more advanced set of GTS allocation schemes, where the coordinator is able to adapt to varying bandwidth requirements of sensor nodes, was shown to improve waiting times and fairness as compared with a naive static method [15,16].

All of the above works focus on application requirements and traffic characteristics when performing slot assignments, rather than the state of the wireless channel, which in a BAN can sometimes be highly volatile yet at other times can have a coherence time of up to 400 ms [17]. More specifically, variable scheduling based on application characteristics tends to focus on the goal of increasing throughput and fairness. In contrast, our work focuses on variable scheduling driven by the wireless channel variations, with a goal of increasing reliability.

A variant of variable scheduling of TDMA slots that accounts for wireless link states has been studied in the context of TDMA-based cellular networks. A good discussion of the framework and survey of proposed algorithms in this context, usually referred to as *opportunistic* scheduling, can be found in [18,19]. In general, opportunistic scheduling techniques select a user that will be allowed to transmit in the next slot and attempt to maximize the overall network throughput, subject to satisfying certain QoS constraints, such as fairness or individual minimum throughput requirements. An extension of the same concept is *opportunistic beamforming*, which may select more than a single user with the best channel to transmit at a given time [20]. However, such opportunistic scheduling approaches are not compatible with BANs, as they require the slave nodes to have their radios turned on at all times and continuously available for polling and/or immediate assignment of the next slot. This requirement is incompatible with *radio duty-cycling*, allowing the radio to be put to sleep when not used—an energy-saving mechanism that is at the core of energy-efficient protocols in BAN [1–6] and sensor networks in general.

## 3. Model and Problem Formulation

### 3.1. System Description

We consider a typical medical application of a Body Area Network, such as monitoring of vital life signs (e.g., blood pressure, heart rate, respiratory rate or temperature) [6]. In this setting, a fixed pre-determined number of sensor nodes are employed around the patient's body. The nodes are generating data at regular time intervals, to be delivered to the hub (e.g., a smartphone) for further processing and/or reporting to a health specialist [21]. Our study focuses on the MAC layer of the nodes, where communication is organised in TDMA rounds (superframes), such that each sensor receives one dedicated time slot per round from the hub, during which it can transmit data without interference from other nodes. The slots in each round are numbered from 1 to *n*, where *n* is the number of sensors. We assume that all slots are of identical duration and that nodes are synchronized and are aware of the slot boundaries. The allocation of slots to nodes may change from one round to the next; it is computed by the hub and conveyed to the nodes inside the periodic beacon packet at the start of each TDMA round. We denote *K*(*i*) to be the slot assigned to node *i* in a given round, 1 ≤ *K*(*i*) ≤ *n*. Each node is assigned exactly one slot in every round, *K*(*i*) ≠ *K*(*j*) for *i* ≠ *j*.

In our scenario, we set the data generation interval to be equal to the length of a superframe; in other words, each sensor node has one new data sample per round, which it transmits to the hub. Once the transmission attempt is completed, the sensor immediately goes to sleep as an energy-saving measure, regardless of the success of the data delivery. Generally, an ARQ mechanism employing acknowledgments from the hub and retransmissions by the sensor could be employed to increase the data delivery success rate. However, we do not consider such a mechanism in this work, as our focus is on the delivery rate improvements that can be achieved through variable scheduling alone, without expending additional energy for retransmissions (the performance of variable scheduling and its benefits in the presence of retransmissions are discussed in our separate study [22]). We stress that, while the sensors do not receive direct feedback on the outcome of their transmissions, the hub still collects and employs this information for scheduling decisions in future TDMA rounds. More specifically, Section 4 considers scheduling strategies that only use the binary outcome ( success of failure) of the transmission from each sensor, while Section 5 discusses scheduling that uses the actual received signal strength value as well.

### 3.2. Channel Model

In order to gain initial insight into the design of efficient variable scheduling, we assume that each wireless link between a sensor node and the hub evolves according to a discrete Markov process, in which each state is classified as either “good” (allowing transmissions to be received successfully) or “bad” (in which no reception is possible). A similar Markov model for wireless links in BAN environments has been suggested by the IEEE BAN Task Group [23] and shown to capture some of the key aspects of the channel dynamics in BANs [24].

We denote the transition probabilities between the bad and the good state and vice versa by *Pu* (up) and *Pd* (down), respectively; thus, *Pu* denotes the probability of moving from a bad state to a good state within a slot interval, and vice versa for *Pd*. We denote by **P** the Markov transition probability matrix, namely,

$$\text{P}=\left[\begin{array}{cc} 1-Pu & Pu\\ Pd & 1-Pd \end{array}\right]$$

If the probability of a link to be in the good state in a certain slot is denoted by *p*(0), then its probability to be good *k* slots later is

(1) $$p(k)≜[1-p(0)p(0)]\cdot {\left(\text{P}\right)}^{k}\cdot {\left[0\;1\right]}^{T}=\frac{Pu}{V}+\frac{p(0)Pd-[1-p(0)]Pu}{V}\cdot {\left(1-V\right)}^{k}$$

where *V* ≜ *Pu* + *Pd* is the sum of the non-diagonal elements of the transition matrix; we subsequently refer to *V* as a *volatility* measure of the link. In addition, we use $S≜\frac{Pu}{V}$ to denote the steady-state probability of the link to be in a good state, to which *p*(*k*) tends as *k* → ∞. We henceforth prefer to characterize the link dynamics using the steady-state probability *S* and volatility *V*, rather than directly using *Pu* and *Pd* (the raw Markov transition probabilities can be computed from *S* and *V* as follows: *Pu* = *S* · *V*, *Pd* = (1 − *S*) · *V*).

For the specific cases where a link is initially known to be in the bad state (*p*(0) = 0) or the good state (*p*(0) = 1), Equation (1) can be simplified further:

(2) $$p(k)=\{\begin{array}{ll} S-S{\left(1-V\right)}^{k} & \text{if}\;p(0)=0\\ S+(1+S){\left(1-V\right)}^{k} & \text{if}\;p(0)=1 \end{array}$$

Note that *p*(*k*) is monotonically increasing (decreasing) if *p*(0) is lower (higher) than the steady-state value *S*. We point out that the same monotonicity property, which will be important in our subsequent analysis, holds in general for Markov processes with multiple states as well.

We emphasize that the simple Markov model outlined above is only used to develop an initial set of scheduling techniques that are evaluated in Section 4, while the strategies in Section 5 are designed and evaluated on experimental RSSI traces [12] directly.

### 3.3. Evaluation Method

In order to obtain a set of realistic ranges for steady state and volatility values, we have used traces from a publicly available data set that contains experimental RSSI measurements from devices strapped to human subjects performing everyday activities over the course of several days [12]. We refer the reader to [25] for further details about the experimental setup. To calculate the parameters *S* and *V* from such traces, it is necessary to set the attenuation outage threshold, *i.e.*, the difference between transmission power and receiver sensitivity. Specifically, if the instantaneous pathloss between a sensor node and the hub is above this threshold then transmitted packets cannot be received (*i.e.*, the corresponding link is in a “bad” state). Since there is no commonly accepted de-facto standard implementation of a BAN radio, our evaluations are performed for a range of attenuation outage thresholds from 75 dB to 95 dB. We point out that with values below 75 dB, wireless links become too unreliable for practical purposes, as will be discussed in the next paragraph. On the other hand, attenuation outage thresholds beyond 95 dB are unlikely to be practical (e.g., for the nominal BAN transmission power of -10 dBm, this would correspond to a radio sensitivity below -105 dBm, which, to the best of our knowledge, is beyond the capability of existing low-power radios). Moreover, as we will show, the exploration of this range can guide radio designers in their decision on choosing the attenuation threshold, based on quantitative data. Additionally, we set the length of each slot to be 5 ms, which is small enough to allow fine-grain control of transmission scheduling but does not introduce excessive overhead from frequent changes of the radio state.

**Figure 1.**
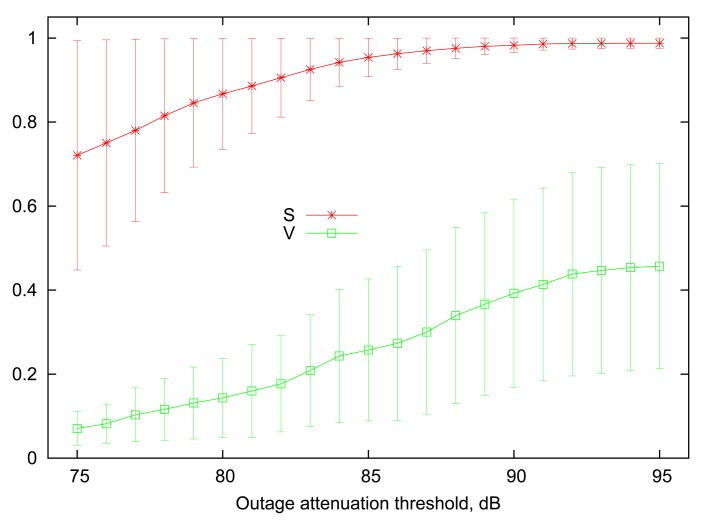
Typical ranges for steady state and volatility.

Figure 1 illustrates 90% percentile ranges of link steady state (*S*) and volatility (*V*) as a function of outage attenuation threshold, computed among the entire set of various on-body links present in the traces. Employing percentile ranges in this context allows to remove outlier links that are either up or down 100% of the time, regardless of the selected threshold. Figure 1 confirms the intuition that increasing the attenuation threshold leads to increased average steady state, *i.e.*, the probability to find a particular wireless link in a “good” state. At the low end of outage threshold values, the differences between the steady states of the links become more pronounced, and several links reach very low values of *S*, making them marginally useful for communication. The link volatility measure follows an opposite trend, where a high attenuation threshold implies that links are more likely to change their state rapidly. More specifically, an outage on any link is unlikely to last more than 10–20 ms when the attenuation threshold is between 90 dB to 95 dB.

For our numerical evaluations in Section 4, the values of *S_i_* and *V_i_* for each link *i* will be drawn randomly from a uniform distribution given by the ranges shown in the Figure 1. Since different values of *S* will be chosen for each individual link, we cannot use the attained reliability (*i.e.*, the fraction of packets successfully delivered) directly as the performance metric, as it depends strongly on the average link uptime. Rather, we focus on the *relative* reliability improvement, or, in other words, the fraction of losses reduced relative to the expected rate of 1 − *S* that can be attained in a system with static scheduling and no retransmissions. For example, if a link has a steady state probability of *S* = 0.9, then it is expected that 10 out of every 100 transmissions on average will fail. If variable scheduling (or a retransmission mechanism) increases the successful delivery rate to 94% of all packets, *i.e.*, losing only 6 out of every 100 packets, then this translates to a relative fraction of 0.4 of losses avoided.

## 4. Simple Slot Assignment Strategies

We now consider the slot assignment strategy by the hub assuming that the wireless links evolve according to the Gilbert model described in the previous section. We define an *optimal* assignment strategy as one that maximizes the long-term throughput from the sensor nodes, or, in other words, minimizes the expected percentage of slots that result in transmission failures. While this definition seems straightforward, the precise formulation of the respective optimization problem is trickier than appears at first glance, as it depends on the information available to the hub. More specifically, if the hub knows the instantaneous states of links to all sensor nodes at the start of each round, then the optimal slot assignment for the next round is simply the permutation that maximizes

(3) $$\underset{K}{\max}\sum_{i=1}^{n}p_{i}(K(i))$$

where *p_i_*(*x*) is the probability of link *i* to be good after *x* slots, as given by Equation (2).

However, while not entirely unrealistic, a full knowledge of all the link states by the hub would incur a significant communication and energy overhead at the start of a TDMA round, as each sensor node would then need to actively sample its wireless channel (e.g., with the transmission of a probe). Accordingly, we are particularly interested in scheduling strategies that only use information already available without additional probing—namely, the outcome (success or failure) of the transmission by each sensor in the previous round. If we denote the number of slots elapsed since the transmission of node *i* by *D*(*i*) (in other words, *D*(*i*) is the amount of time that the information about the link state of node *i* is outdated), then the expected number of successful transmissions in the next round is given by:

(4) $$\sum_{i=1}^{n}p_{i}(D(i)+K(i))$$

It is interesting to note that repeated slot assignments seeking to maximize (4) in every round may not necessarily lead to the long-term optimal performance. This is due to the fact that the information available to the hub depends on the scheduling decisions taken in the previous round; and hence we refer to a permutation that maximizes (4) as a *single-round* optimal assignment.

For the initial consideration of the optimal scheduling strategies, we assume that the statistical parameters *S* and *V* of each link are known to the hub. However, we will return to this point later, and show that in fact one of the proposed strategies can be applied even without this knowledge and still achieve near-optimal performance in most scenarios of interest.

### 4.1. Scheduling Based on Full State Information

We initially consider the optimal scheduling under the assumption that full information about current channel states is available to the scheduling algorithm at the start of each TDMA round. As explained above, this can be achieved in theory by probing all the links before making a scheduling decision, leading, however, to an unacceptably high overhead in terms of both time and energy costs it imposes on the sensor nodes. We therefore emphasize that the assumption of full information is only used in this subsection to obtain an upper bound reference, and it will be alleviated thereafter.

If the channel state information is always available to the scheduler, then the optimal long-term performance is achieved simply by maximizing the rate of successful transmissions at each round independently. Thus, the optimization target is given by Equation (3). To that end, we observe that finding the permutation *K* that maximizes (3) can be seen as an instance of the *maximum-weight matching* problem in bipartite graphs. Indeed, define a bipartite graph with *n* vertices corresponding to the sensor nodes, and a further *n* vertices corresponding to the time slots. Define an edge from node *i* to slot *j* to have a weight of *p_i_*(*j*), *i.e.*, the probability for the node's link to be in a good state in that slot. Then a scheduling assignment that maximizes the expected number of successful transmissions (3) is equivalent to a maximum-weight matching in the corresponding bipartite graph.

We point out that well-known algorithms for solving the maximum-weight matching problem exist, requiring *O*(*n*^3^) time in general [26,27]. However, such a complexity cannot be considered reasonable for more than a very small number of nodes (in fact, the standard allows up to 256 nodes [11]), since the solution is required essentially instantly. Indeed, any delay in the scheduling computation translates directly to a delay in the start of the next TDMA round. Accordingly, we are much more interested in very low-complexity heuristics to approximate the optimal scheduling solution, not exceeding that of a simple sorting operation.

To that end, we proceed by establishing a basic property of the optimal solution.

#### Lemma 1

If *G* is the subset of links in the “good” state at the start of the round, i.e., *G* = {*i*|*p_i_*(0) = 1}, then in the optimal scheduling K*, K*(*i*) < K* (*j*) for any *i* ∈ *G* and *j* ∉ *G*.

Lemma (1) captures the intuition based on a fundamental property of the function *p*(*k*) (which holds more generally in Markov chains with multiple states as well), namely, that if a probability of a given state at some time is higher (lower) than its steady-state value, then it will monotonically decrease (increase) towards that steady-state value. Consequently, it is best to bring the links initially known to be “good” forward to have their transmissions as early as possible, while deferring all “bad” links to the end of the round. However, the question of ordering within each of the subsets remains, and unless all links have identical Markov transition probabilities, the expected number of successful slots will depend on the chosen order within each subset.

Accordingly, we define the following scheduling approaches that will be evaluated further:

1. *Random Groups*: Schedule all nodes with “good” links at the start of the round first, followed by “bad” ones; within each subset, schedule the transmissions randomly (*O*(*n*) complexity).
2. *Greedy Sorting*: For each unassigned slot *j*, starting with *j* = 1, compute the value of *p*′(*j*) ≜ *p*(*j*) − *p*(*j* + 1) for all nodes not yet allocated; then assign slot *j* to the link with the highest value of *p*′(*j*) (*O*(*n*^2^) complexity).
3. *Optimal*: Compute the optimal schedule using an algorithm for solving the maximum-weight bipartite matching (*O*(*n*^3^) complexity).

The rationale behind the *Greedy Sorting* heuristic is that $p_{i}^{\prime }(j)$ represents the “loss” of success probability if the assignment of link *i* is deferred from slot *j* to slot *j* + 1. Therefore, for example, it makes sense to assign to the first slot the link that would cause the highest reduction of the target expression (3) if pushed back by one slot; a similar reasoning then applies to the subsequent slots. This heuristic may not lead to the optimal scheduling in general, since the order among *p*′(*j*) may change as *j* progresses (links with a higher volatility *V* converge to their steady state probability quicker, and thus have a high *p*′(*j*) when *j* is small yet low *p*′(*j*) later). However, *Greedy Sorting* has the advantage of a lower complexity than the optimal assignment algorithm, and in many cases achieves a comparable performance.

**Figure 2.**
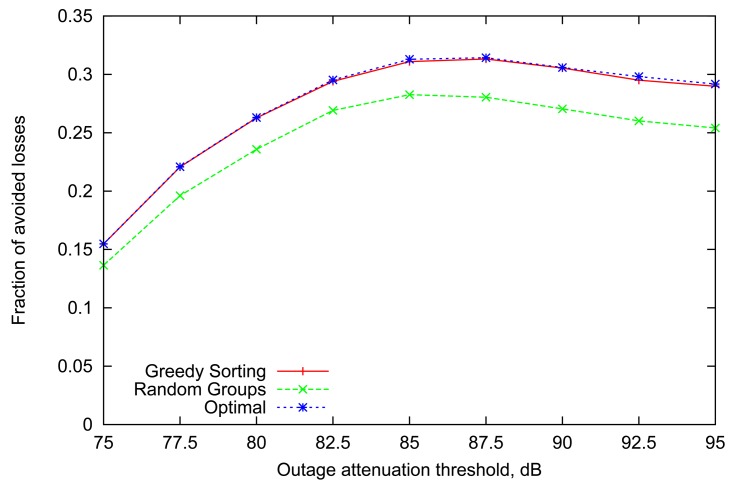
Performance based on link state information at the start of each round.

Figure 2 presents the relative loss rate reduction obtained by simulating the above strategies with a representative number of 8 sensor nodes (*n* = 8) in a BAN, similar to the setup described in [8]. The general trend is very consistent across all values of outage attenuation threshold. More specifically, the performance of the *Greedy Sorting* algorithm is almost identical to *Optimal*, despite its lower complexity. *Random Groups* scheduling lags behind due to differences in the links' dynamic parameters which are not accounted for (indeed, Random Groups is optimal only for homogeneous links).

It is interesting to point out that the gain achieved by variable scheduling declines for extreme values of outage threshold. Indeed, when the threshold is low, the average volatility and steady state of all links are also low (as it was shown in Figure 1), thus outages happen frequently and last longer, often resulting in several consecutive failures over the course of multiple rounds, obviously making any scheduling decisions less relevant. A slight decline for high values of attenuation threshold can also be noted, where both the steady state and volatility of all links is high. Although the effect will be more pronounced with the scenario in the next subsection, the underlying reason is that highly volatile links imply that information about the state of a wireless link becomes less useful after only a few slots (*i.e.*, converging rapidly to the steady-state average).

### 4.2. Scheduling Based on Last Round Outcomes

As explained earlier, requiring the nodes to actively sample their channels at the start of each round would impose an unacceptably high overhead, in terms of both time and energy. We now move away from that assumption and discuss scheduling algorithms that rely only on the outcome of the communication during the previous round, which is available anyway and does not require additional effort to obtain.

We first point out that, despite the delayed information, it is still true that the probability of being in a good state continues to monotonically decrease over time for any link that was known to be good in the previous round, and, conversely, monotonically increase for any link that was bad. Consequently, the result of Lemma (1) continues to hold; in other words, it is still always better to schedule all the links in the “good” group ahead of the “bad”. However, it is no longer true that, if all the links have identical Markov transition parameters, then the ordering within each group does not matter. Our next result explains this fact in detail and presents the optimal transmission schedule in this case.

#### Lemma 2

#### Consider a system with *n* links with identical transition probabilities (*S_i_* = *S_j_* and *V_i_* = *V_j_* for all 1 ≤ *i*, *j* ≤ *n*), and denote by *K*′(*i*) the slot assigned to link *i* in the previous round. Denote *G* as the subset of links observed in the “good” state during their allocated slot in the previous round, i.e., *G* = {*i*|*p_i_*(0) = 1}. Then, for any *i*, *j* such that *K*′(*i*) > *K*′(*j*), the following properties hold for the single-round optimal allocation K*

1. K*(*i*) < K*(*j*) *if i* ∈ *G and j* ∉ *G*;
2. K*(*i*) > K*(*j*) *if i* ∉ *G and j* ∈ *G*;
3. K*(*i*) < K*(*j*) *if i*, *j* ∈ *G*;
4. K*(*i*) > K*(*j*) *if i*, *j* ∉ *G*.

In other words, the order of the links in the “good” group should be reversed while the order of the links in the “bad” group should be kept the same as in the last round.

As a result of Lemma (2), we add another strategy to our repertoire: schedule all links that were good in the previous round ahead of the bad ones; and within the good group only, invert the order of the transmissions from the last round. We refer to this approach as the *Flipping* strategy, since for a given set of links, the order of their transmissions will be “flipped” among consecutive rounds as long as they are successful (which is the most common outcome when the link steady state values are high). While the *Flipping* strategy is optimal only in a system with homogeneous links, it has several other desirable characteristics—specifically, it is trivially easy to compute and it does not require the knowledge of the Markov parameters of the individual links. Thus it becomes interesting to apply it as a heuristic in non-homogeneous scenarios, *i.e.*, with diverse channels whose Markov parameters take a range of different values.

**Figure 3.**
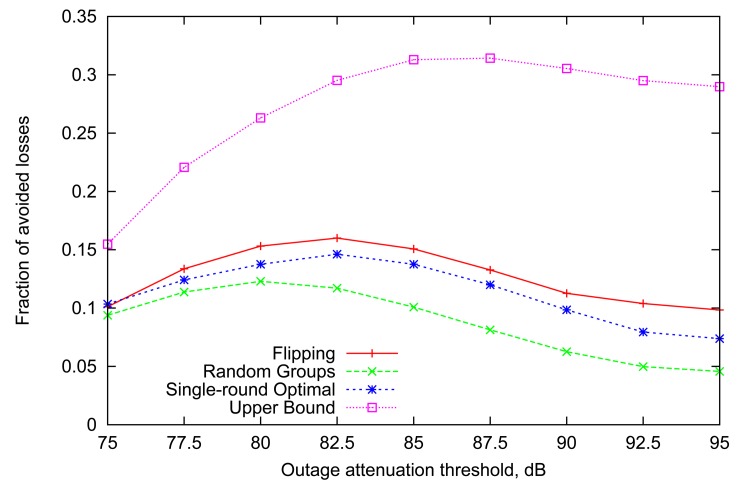
Performance based on link state information from the previous round.

The graph in Figure 3 depicts the performance of different variable scheduling strategies together with the maximum performance achieved in the previous subsection (shown as the *Upper Bound* in this graph). We note that although the gains are much lower since the information available is more outdated, the general trends seen earlier persist here as well. Furthermore, the decline of performance for high values of outage threshold is more pronounced since highly volatile links make outdated information even less useful.

However, the most interesting finding is that the performance of the newly introduced *Flipping* strategy is not only comparable with, but in fact is even consistently better than that of the single-round optimal algorithm for almost all values of the outage threshold. This happens despite the fact that optimal assignment accounts for individual Gilbert parameters of each link, while *Flipping* strategy relies solely on the outcomes of transmissions in the preceding round. We emphasize that the underlying Markov parameters are not homogeneous across all links, but (for each link independently) drawn from a range of typical values, as explained in Section 3. We discuss the insights behind this counter-intuitive performance effect in the next subsection.

### 4.3. Long-Term Performance

As mentioned earlier, when the scheduling is not based on the instantaneous channel state information at the start of the round, it is no longer necessarily true that a scheduling strategy that maximizes the expected number of successful links in each round will also achieve the best *long-term* performance. This is because the schedule choice in a given round has an indirect effect on the information that will be available to the scheduler at the next round. Thus, a certain schedule may maximize the expected number of successful links in the upcoming round but leave the scheduler with “inferior” information, in some sense to make its choice in the subsequent round.

Consider the following artificial example to illustrate this effect. Assume *n* links where all links but one have their steady states equal to 1, thus making their probability of successful transmission, *p*(*k*), always 1 regardless of the value of *k*. Consecutively, slot assignment strategies focus around the state of the single link that does not have perfect reception. Since both Flipping and Single-round optimal strategies would choose to place the transmission on that link as late as possible in the round if a failure happens, we focus on the situation where a number of consecutive successful transmissions occur over the course of several rounds. Based on the monotonicity property of *p*(*k*) shown earlier, the single-round optimal allocation would always schedule the transmission in the earliest possible slot, as any increase in the value of *k* leads to decrease in *p*(*k*) given that *p*(0) = 1. Thus, the transmission of this variable link will be assigned to slot 1 as long as consecutive successes occur. This means that there will always be exactly *n* slots between those consecutive transmission attempts and the Single-round optimal allocation will have approximately *p*(*n*) × *l* successful transmissions on that link individually over the course of *l* rounds. The Flipping strategy would place the node's transmission at the first slot in one round and at the last slot in the following round, repeating that pattern as long as consecutive successes occur. Thus the interval between transmissions would alternate between 1 and 2*n* − 1 slots, making the probability of successful transmission *p*(1) and *p*(2*n* − 1) respectively. Note that while *p*(2*n* − 1) < *p*(*n*) (making such assignment not optimal for the round), but *p*(1) > *p*(*n*), in other words, the Flipping algorithm sacrifices in one round but gains in the following round, if compared with the single-round optimal schedule. As it turns out, for most practical combinations of link parameters *S* and *V* , the gain achieved by Flipping the slots significantly outweighs the loss caused by not following the optimal slot assignment in every second round, as was indeed shown by the numerical evaluation results. An in-depth discussion on this long-term performance effect, as well as an exhaustive evaluation of a two-node scenario showing how close the *Flipping* strategy is to the long-term optimal schedule, can be found in [28].

## 5. Scheduling Based on RSSI Information

The previous section explored scheduling strategies based only on the binary outcome (*i.e.*, success or failure) of the most recent transmission on each link. However, this does not exhaust the information available to the network coordinator. In particular, the RSSI value itself is a useful benchmark of the quality of a wireless link, which is indeed used in many practical algorithms for transmission power control and routing. In this section, we investigate how the RSSI reading can be used to make informed scheduling decisions and compare the results with the strategies proposed earlier, that rely purely on the binary outcome information.

Since the Gilbert model discussed in Section 3 does not capture the fluctuations of RSSI value, in this section we move away from any particular model and evaluate the scheduling strategies using the RSSI traces directly. We emphasize that the design of our algorithms is still based on the same insights from previous sections, *extending* the functionality to account for RSSI dynamics rather than creating a whole new set of scheduling strategies tailored to a specific set of experimental traces.

**Figure 4.**
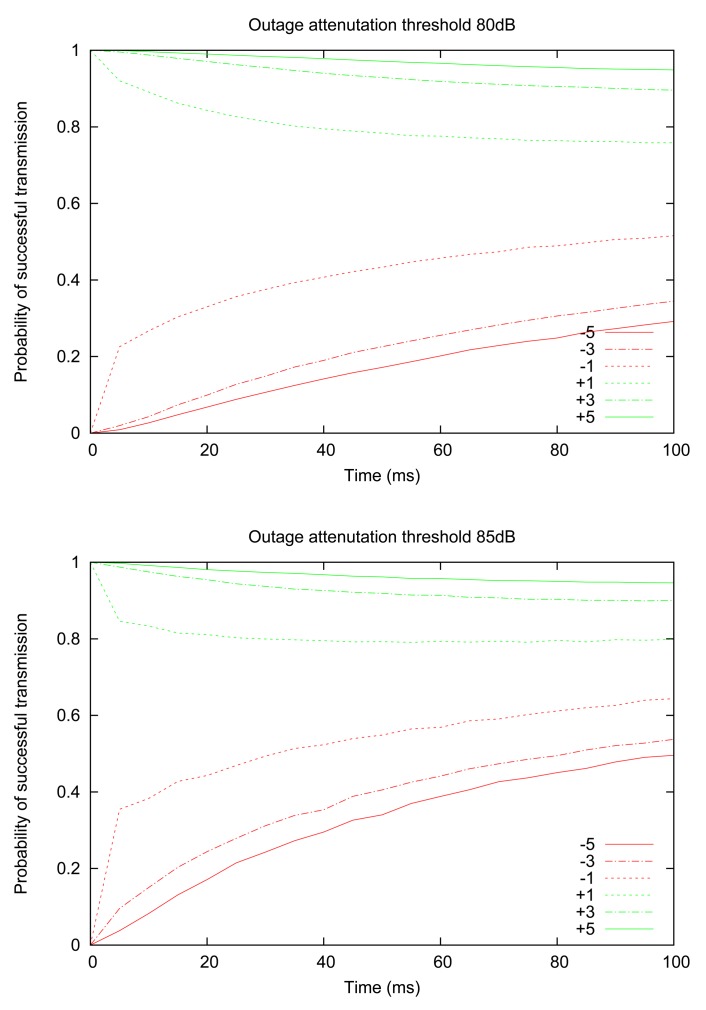
Probability of successful transmission based on most resent RSSI reading, with attenuation thresholds of 80 dB and 85 dB.

We proceed by examining how the probability of successful transmission evolves over time for different RSSI values, thus establishing a connection with the insights gained in the previous section. Figure 4 shows the experimentally computed probability of successful transmission, for two values of outage thresholds (80 dB and 85 dB), starting from an initially observed RSSI value that is above or below the outage threshold by a given margin (e.g., “+5” means that the initial RSSI measurement is 5 dB above the threshold value). The graph illustrates that the central claim of the previous section continues to hold; namely, the probability of successful transmission still decreases monotonically for “good” links (*i.e.*, initially observed RSSI is above the threshold) and increases monotonically for “bad” links. An additional insight provided by Figure 4 is that when the initial RSSI value is close to the threshold, the probability converges to a long-term value much more rapidly; in other words, we verify the intuition that links with RSSI readings close to the threshold are more volatile (*i.e.*, more likely to cross the threshold).

### 5.1. Scheduling Based on Full State Information

Once again, we start with the simple case where the information (namely, RSSI readings) from all links is available to the scheduler at the beginning of the round. Recall that in the corresponding scenario based on the Gilbert model, we introduced three scheduling strategies: *“Random Groups”*, *“Greedy Sorting”*, and *“Optimal”*. While the “Random Groups” algorithm can be applied in a straightforward manner here as well, the other two strategies require the knowledge of the Gilbert model parameters *S_i_* and *V_i_* of individual links, and cannot be applied on experimental traces directly. However, we can still employ the rationale behind the *“Greedy Sorting”* algorithm, which dictates that more volatile good links should be scheduled as early in the round as possible, as their probability to have a successful transmission decays more rapidly. From the previously established connection between volatility and proximity of the RSSI value to the threshold, we introduce the *Greedy RSSI Sorting* algorithm, as follows: schedule all “good” links before all “bad” links, and within the “good” group, sort the links by RSSI value in increasing order.

**Figure 5.**
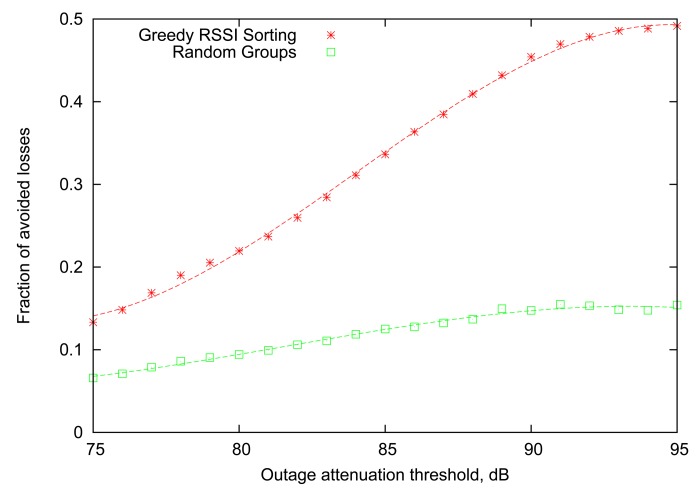
Performance based on RSSI reading at the start of each round.

Figure 5 compares the performance of the *Greedy RSSI Sorting* algorithm with the baseline provided by *Random Groups*. It can be seen that smart ordering of transmissions within the “good” group based on RSSI information provides a clear advantage for all values of attenuation threshold, reaching more than 45% reduction in losses for attenuation thresholds of 90 dB to 95 dB. Another important observation is that the performance of the *Random Groups* algorithm is noticeably lower than that in Figure 2, illustrating that the Gilbert link model is, in fact, only an approximation of the real behaviour of the body-area links.

### 5.2. Scheduling Based on Last Round Outcomes

**Figure 6.**
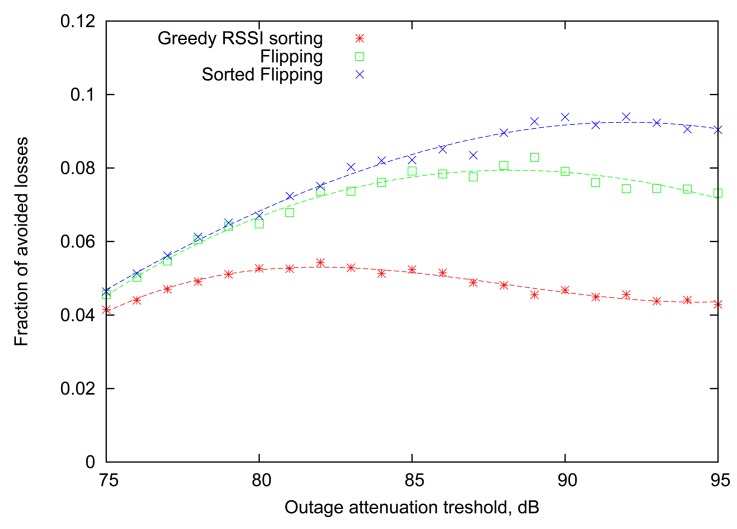
Performance based on RSSI reading from the previous round.

As the next step, we aim to find out how the newly introduced *Greedy RSSI Sorting* algorithm will behave in the more realistic situation, when the scheduling is based on the outcomes of transmissions in the previous round. For this case, the *Flipping* strategy will serve as the baseline, indicating the attainable loss rate reduction if the RSSI information is not taken into account. This comparison is performed in Figure 6. As it turns out, the *Greedy RSSI Sorting* strategy lags significantly behind *Flipping* in performance. This can be explained by the fact that the *Greedy RSSI Sorting* algorithm allocates transmission slots focusing greedily on maximizing the expected number of successes in the current round only and ignoring the impact on subsequent rounds. Thus, it suffers from the same long-term effect that causes *Flipping* to perform better than the single-round *Optimal* strategy in Section 4.

Despite the above fact, the RSSI information can still be useful for scheduling decisions based on the previous round outcomes, and we devised a strategy to combine the strengths of *Flipping* and *Greedy RSSI Sorting*. The main benefit of *Flipping* comes from the long-term effect, where sacrificing some of the performance in the current round allows more gains in the next round (by having more recent link state information). On the other hand, *Greedy RSSI Sorting* algorithm considers short-term benefits by prioritizing highly volatile links. Thus, we define the “RSSI-sorted Flipping” (or, for brevity, *Sorted Flipping*) algorithm as follows.

Similar to our previous strategies, the hub defines two groups of nodes: those that should be scheduled as early in the round as possible (the “Early” group), and, conversely, as late in the round as possible (the “Late” group). Previously, the “Late” group corresponded to the subset of nodes with bad links and the “Early” group only contained those with good links, but now we allow more flexibility. More specifically, the steps taken by the *Sorted Flipping* strategy are as follows:

1. Initially, the nodes are equally split between the two groups in a random manner.
2. Every round, each node is moved to the opposite group unless it failed to transmit in the previous round, in which case it is forced to the “Late” group.
3. Slots are assigned by increasing RSSI order in the “Early” group and by decreasing order in the “Late” group. If a failure happened in the previous round, that node is assigned a large negative RSSI value, implying that it will be scheduled as late as possible.

Note that if the RSSI reading of each node remains constant across consequent rounds, then the algorithm will behave exactly as simple *Flipping*. From Figure 6 it is evident that *Sorted Flipping* consistently improves the performance of *Flipping*, and the gain is most pronounced for large values of the attenuation outage threshold.

## 6. Conclusions

To improve the reliability in Body Area Networks, we have presented a framework for variable TDMA scheduling, where transmission slots are assigned to nodes based on the information about their wireless links, with the goal of minimizing transmission failures due to bad channel state. Based on a two-state Gilbert link model we have developed the simple yet effective *Flipping* strategy, which creates an optimal slot allocation for a single TDMA round when the links of all nodes are identical. Additionally, *Flipping* has several other desirable characteristics, namely it is trivial to compute and performs well in practical scenarios, where wireless links of the sensor nodes are not homogeneous.

Furthermore, we have conducted additional evaluations directly on an experimental set of RSSI traces, reinforcing the main scheduling principles and extending the *Flipping* strategy to account for RSSI information, making more informed scheduling decisions. It is important to emphasize that, while these strategies were inspired by a two-state Markov channel model, their operation is based only on the outcome of each transmission directly (*i.e.*, the success/failure of the transmission, or the measured RSSI value), and does not require the *a priori* knowledge of any statistical parameters of the links. We have shown that up to 45% of all losses caused by bad channel state, can be avoided simply by smarter allocation of TDMA slots if instantaneous channel state is available; or, in a more realistic setting where only the previous round outcomes are used for scheduling purposes, up to 10% of all transmission failures can be prevented.

We highlight that all the techniques proposed in this paper operate by varying the transmission schedule of sensor nodes only, and the performance improvements are achieved without consuming any additional energy (e.g., by retransmissions) or increasing the latency of incoming packets. It is worth noting that in a separate work [22] we have shown that variable scheduling techniques continue to perform well when retransmissions are employed to further increase reliability. More specifically, if the retransmission mechanism follows the same scheduling principles, taking into the account the wireless link state information of sensor nodes, then the reliability metric can be further improved for the same amount of extra energy spent on retransmissions.

Our study has aimed to demonstrate the benefits of variable scheduling at a proof-of-concept level. There are many practical issues that remain to be addressed in the design of a full MAC protocol incorporating the ideas of this paper and [22] (e.g., the structure of a beacon packet, overcoming losses of management packets, *etc.*). The implementation of such a MAC protocol in a simulation platform (Castalia) and in real devices is the subject of ongoing work.

## Appendix

### Proof of Lemma 1

Consider an allocation *K* that violates the lemma claim, *i.e.*, such that *K*(*i*) > *K*(*j*) for some *i* ∈ *G* and *j* ∉ *G*. Using the definition of *p*(*k*) (Equation ((2))), we calculate the change in the target value (3) if the assignments of *i* and *j* are swapped. We do so in two steps, first computing the change *p_i_*(*K*(*j*)) − *p_i_*(*K*(*i*)) from moving link *i* to slot *K*(*j*), and then similarly the effect of moving link *j* to slot *K*(*i*).

(5) $$p_{i}(K(j))-p_{i}(K(i))=(1-S_{i})\cdot ({\left(1-V_{i}\right)}^{K(j)}-{\left(1-V_{i}\right)}^{K(i)})>0;$$

(6) $$p_{j}(K(i))-p_{j}(K(j))=S_{j}\cdot ({\left(1-V_{j}\right)}^{K(j)}-{\left(1-V_{j}\right)}^{K(i)})>0;$$

We observe that both links improve their success probabilities as a result of the swap. Consequently, the assignment *K* cannot be optimal.

### Proof of Lemma 2

The first and second claims of the lemma are essentially a repetition of Lemma (1) and follow trivially from the monotonicity of *p_i_*, *p_j_*.

Consider an allocation *K* that violates the third claim of the lemma, *i.e.*, *K*(*i*) > *K*(*j*) and *K*′(*i*) > *K*′(*j*) for some *i*, *j* ∈ *G*. The probability of link *i* to be successful in the next round is then given by *p_i_*(*n* − *K*′(*i*) + *K*(*i*)), where *p_i_* is defined by the second case of (2); and similarly *p_j_*(*n* − *K*′(*j*) + *K*(*j*)) for link *j*. We now calculate the gain in the optimization target (4) if the assignments of *i* and *j* are swapped:

(7) $$[p_{i}(n-K^{\prime }(i)+K(j))+p_{j}(n-K^{\prime }(j)+K(i))]-[p_{i}(n-K^{\prime }(i)+K(i))+p_{j}(n-K^{\prime }(j)+K(j))]=(1-S_{i}){\left(1-V_{i}\right)}^{n-K^{\prime }(i)+K(j)}+(1-S_{j}){\left(1-V_{j}\right)}^{n-K^{\prime }(j)+K(i)}-(1-S_{i}){\left(1-V_{i}\right)}^{n-K^{\prime }(i)+K(i)}-(1-S_{j}){\left(1-V_{j}\right)}^{n-K^{\prime }(j)+K(j)}$$

Since the parameters of all links are identical, we can denote *S* = *S_i_* = *S_j_*, *V* = *V_i_* = *V_j_*, and obtain

(8) $$(1-S)\left[{\left(1-V\right)}^{n-K^{\prime }(i)+K(j)}-{\left(1-V\right)}^{n-K^{\prime }(i)+K(i)}+{\left(1-V\right)}^{n-K^{\prime }(j)+K(i)}-{\left(1-V\right)}^{n-K^{\prime }(j)+K(j)}\right]=(1-S)\left[{\left(1-V\right)}^{n-K^{\prime }(i)}-{\left(1-V\right)}^{n-K^{\prime }(J)}\right]\cdot {\left[{\left(1-V\right)}^{K(j)}-(1-V\right)}^{K(i)}]>0$$

since *n* − *K*′(*i*) < *n* − *K*′(*j*) and *K*(*j*) < *K*(*i*). This shows that swapping the allocations of *i* and *j* improves the target value (4); consequently, *K* cannot be the optimal scheduling.

The proof of the fourth lemma claim follows a very similar derivation to the above, and is omitted for brevity.
